# Phase II trial of hypofractionated VMAT-based treatment for early stage breast cancer: 2-year toxicity and clinical results

**DOI:** 10.1186/s13014-016-0701-z

**Published:** 2016-09-17

**Authors:** Fiorenza De Rose, Antonella Fogliata, Davide Franceschini, Piera Navarria, Elisa Villa, Cristina Iftode, Giuseppe D’Agostino, Luca Cozzi, Francesca Lobefalo, Pietro Mancosu, Stefano Tomatis, Marta Scorsetti

**Affiliations:** 1 Radiotherapy and Radiosurgery Department, Humanitas Research Hospital and Cancer Center, Milan-Rozzano, Italy; 2 Department of Biomedical Sciences, Humanitas University, Milan-Rozzano, Italy

**Keywords:** Breast cancer, Simultaneous integrated boost, Hypofractionation, Volumetric modulated arc therapy

## Abstract

**Background:**

To report toxicity and early clinical outcomes of hypofractionated simultaneous integrated boost (SIB) approach with Volumetric Modulated Arc Therapy (VMAT) as adjuvant treatment after breast-conserving surgery.

**Methods:**

Patients presenting early-stage breast cancer were enrolled in a phase II trial. Eligibility criteria: age > 18 years old, invasive cancer or ductal carcinoma in situ (DCIS), Stage I-II (T < 3 cm and N ≤ 3), breast-conserving surgery without oncoplastic reconstruction. Any systemic therapy was allowed in neoadjuvant or adjuvant setting. All patients underwent VMAT-SIB technique to irradiate the whole breast and the tumor bed. Doses to whole breast and surgical bed were 40.5 Gy and 48 Gy, respectively, delivered in 15 fractions over 3 weeks. Acute and late skin toxicities were recorded. Cosmetic outcome was assessed as excellent/good or fair/poor.

**Results:**

The present study focused on results of a cohort of 144 patients with a minimum follow-up of 24 months (median 37, range 24–55 months). Median age was 62 years old (range 30–88). All patients had an invasive carcinoma (no patients with DCIS were present in this subset). At one year, the highest reported skin toxicity was G1, in 14 % of the patients; this data dropped to 4 % at the last follow-up, after more than 2 years. Breast pain was recorded in 21.6 % of the patients 6 months after treatment, while it was present in 3.5 % of the patients at the last follow-up, showing a significant improvement with time. Correlation between liponecrosis and boost target volume was found not significant. Breast pain was correlated with breast volume. No pulmonary or cardiological toxicities were recorded. After an early evaluation of clinical outcomes, only one case presented disease relapse, as liver metastases.

**Conclusions:**

The 3-week VMAT-SIB course as adjuvant treatment after breast-conserving surgery showed to be well tolerated and was associated with optimal local control. Long-term follow-up data are needed to assess late toxicity and clinical outcomes.

## Background

Incidence of early stage breast cancer increased in the last decade thanks to early diagnosis and screening programs. The treatment approach with breast conserving surgery (BCS) followed by whole-breast radiotherapy (WBRT) has been shown to be equivalent to mastectomy in terms of local control and survival [1–3]. Traditionally, conventional fractionation schedules for radiotherapy give 50 Gy in 2 or 1.8 Gy daily fractions, often with an additional sequential boost to the tumor bed, resulting in the overall treatment time (OTT) of 6–7 weeks [4]. Recently, there has been a shift in clinical studies toward the delivery of adjuvant radiotherapy using shorter treatment schedules. Clinical data showed that breast cancer might present α/β value around 4 Gy similar to the late-reacting healthy tissues, suggesting the possible benefit of hypofractionation in breast cancer treatment [5]. These radiobiological points [6] inspired phase III randomized trials comparing standard schedule to different hypofractionated schemes using larger doses per treatment, resulting in shorter OTT.

All published trials have used schedules involving 13 to 16 treatment sessions to the whole breast over 3 to 5 weeks, with more than 6500 patients enrolled. Ten-year data from these studies (Royal Marsden trial, Canadian trial, and the START A and B trials) demonstrated that hypofractionation is associated to equivalent or improved outcomes (both local and distant disease control), toxicity, cosmesis, and cost-effectiveness [7–11]. Moreover, shorter treatment time results also in greater patient convenience and resource efficiency.

Consequently, in 2013, the American Society for Radiation Oncology (ASTRO) released a recommendation to strongly consider the use of shorter treatment schedules in the radiotherapy adjuvant treatment for women age ≥ 50 years old with early stage invasive breast cancer after BCS. This is one of the “Choosing Wisely” recommendations, as part of a campaign by the American Board of Internal Medicine Foundation to encourage the choice of evidence-based treatments (http://www.choosingwisely.org/clinician-lists/american-society-radiation-oncology-whole-breastradiotherapy/).

Unfortunately, there are still open issues about the use of hypofractionated radiotherapy in early stage breast cancer. Groups most debated are as follows: young patients, patients with large breasts, patients undergoing chemotherapy (neoadjuvant or adjuvant). Another unsolved question is the association with sequential or concomitant boost [12].

As previously specified, ASTRO guidelines recommended hypofractionated radiotherapy only for patients older than 50 because of a greater risk of local recurrence and distant metastases in younger patients. Despite this recommendation, the Canadian trial confirmed the equivalence in efficacy to prevent local recurrence for both conventional and hypofractionated schemes among younger women, that represented the 25 % of the study population [7]. Moreover, the British agency NICE (National Institute for Health and Clinical Excellence) considers schedules of 50 Gy in 25 fractions or 40.05 Gy in 15 fractions as standard radiotherapy regardless of age at diagnosis [13]. As regards large breast patients, the possible dose heterogeneity associated with large breast volumes in a hypofractionation schedule could potentially worsen the cosmetic outcomes. At last, there are few consistent data concerning aesthetic results and skin toxicity in patients receiving either adjuvant chemotherapy (including agents with known cardiotoxic effects) or tumour bed boost [14, 15].

Recently, technical developments in radiation oncology, such as the use of intensity-modulated radiotherapy, and breathing-adaptive therapy are ongoing to optimize the dose distribution for target dose homogeneity and organs at risk (OARs) sparing.

In this frame, the introduction in the clinical practice of the volumetric modulated arc therapy (VMAT) technique which by optimizing multileaf (MLC) shapes, dose rate and gantry rotation speed [16], allows a general improvement of organs at risk sparing, high target coverage and dose homogeneity, reduced beam-on time and relative low number of monitor units. In addition, the breast cancer treatment has been explored for VMAT delivery [17–20].

Since 2009 the use of VMAT, in its RapidArc form (Varian, Palo Alto, California, USA) was made available in our clinical practice for various tumour sites. From September 2010, an institutional Phase I-II trial on hypofractionated early stage breast irradiation with simultaneous integrated boost (SIB) and RapidArc technique started to recruit patients. The aim of our current work is to present the early toxicity and cosmetic results for patients with at least 2-year follow up enrolled in this prospective Phase I-II trial, who were treated using VMAT and SIB as adjuvant breast cancer radiotherapy.

## Methods

Patients with early-stage breast carcinoma who underwent conservative surgery were enrolled in an institutional phase I-II prospective non-randomized trial of adjuvant radiotherapy with SIB delivered with RapidArc technology (VMAT-SIB). The study received the approval by the Ethical Review Committee, in compliance with the Helsinki declaration. Informed consent was obtained from all individual patients.

As per today, 840 patients have been treated according to the protocol. The primary endpoint of the trial was the evaluation of the feasibility of VMAT-SIB hypofractionation. In 2012, data related to the first 50 treated patients were published [19] proving the technical feasibity, in association with an acceptable acute skin toxicity, similar to what was reported in literature. The secondary endpoint was the evaluation of the toxicity in terms of acute and late side effects. In the current report, we analysed the results concerning this secondary endpoint, focusing only on the sub-group of 144 patients having at least two-year follow-up. The local control was also reported, even if it was not an explicit objective of the study.

Protocol eligibility criteria were: age > 18 years old, invasive cancer or ductal carcinoma in situ (DCIS), AJCC Stage I-II (T-size ≤ 3 cm, N ≤ 3), breast-conserving surgery without oncoplastic reconstruction, and any systemic therapy (neoadjuvant or adjuvant). Radiotherapy treatment started within 60 days from the surgery; if adjuvant chemotherapy was administered, radiotherapy started after 4 weeks from the last chemotherapy cycle. Patients with DCIS were made eligible by an amendment to the protocol since 2013.

For radiotherapy treatment, all patients were set-up in supine position, with both arms above the head. CT dataset was acquired with 3 mm thick adjacent slices. No respiratory gating was adopted.

The clinical target volume CTV of the whole breast was the entire mammary gland. CTV of the boost was the surgical bed, defined by adding 1 cm to the surgical clips placed in the lumpectomy cavity during surgery. Three radiation oncologists took part in the contouring, following common internal delineation guidelines, and no interobserver variability in the target definition was here evaluated. Planning target volumes PTV were contoured by adding a 5 mm margin to each CTV; PTVs were however limited to 4 mm within the skin surface, and excluded ribs and lung parenchyma. The whole breast PTV (PTV_WB) excluded the boost PTV (PTV_boost).

The treatment dose was prescribed with SIB as 40.5 Gy to the PTV_WB and 48.0 Gy to the PTV_boost, in 15 fractions over 3 weeks, delivering 2.7 and 3.2 Gy/fraction to each PTV [19].

Plan objectives were the following: target coverage and homogeneity: near-to-minimum dose D_98%_ > 95 % for both PTVs, near-to-maximum dose D_2%_ < 107 % for PTV_WB. Concerning organs at risk [19]: ipsilateral lung should receive mean dose <10Gy, and the volume receiving more than 20Gy should not exceed 10 % (V_20Gy_ < 10 %); for heart V_40Gy_ < 3 % and V_18Gy_ < 5 %, no specific requests for mean heart dose; minimize contralateral lung and breast irradiation; ribs maximum dose not exceeding 50Gy; skin dose not exceeding 40Gy for cutaneous desquamation: skin dose was recorded for 5 mm thickness of the first skin layers in a region covering the whole breast plus an additional margin of 3 cm around the mammary gland.

Plans were optimized for RapidArc delivery, with two partial arcs in a range from the classical medial tangential beam to the posterior entrance, through the PTV side; PRO algorithm was used to modulate MLC shape and beam intensity during the gantry rotation. The strategy described by Nicolini et al. [21] to have the skin flash was adopted. Dose calculations used the Anisotropic Analytical Algorithm (AAA). Delivery was on 6MV beams from Varian Clinac, Unique or TrueBeam, equipped with a Millennium MLC-120.

To verify the patient positioning before each treatment session, daily CBCT (or 2D-2D matching for the patients treated on Unique linac, not equipped with CBCT) were acquired; eventual shifts as required by CBCT vs. planning-CT co-registration were applied.

Patient clinical evaluation was assessed during the treatment once a week. Follow-up was then scheduled at the end of radiotherapy, at 1, 3 and 6 months after radiation treatment, and then every 6 months for the first 2 years. Hematologic exams (i.e. CBC, liver and renal function, tumour marker Ca15.3), as well as breast ultrasound were scheduled every 6 months, while bilateral mammography every 12 months.

Skin toxicity was visually assessed by objective clinical exam and pictures of the irradiated breast taken in frontal and lateral views during each visit (during treatment and follow-up). This photographic documentation was compared with the baseline performed before the beginning of the radiation treatment; acute toxicity was scored according to RTOG acute radiation morbidity scoring criteria, and late toxicity (from 6 month after RT) according to CTCAE v.4. As late skin toxicity the main endpoint is the hyperpigmentation; fibrosis and teleangiectasia are also reported. Cosmetic outcomes were ranked as: excellent/good vs. fair/poor, according to the Harvard scale [22]. Two observers (a dedicated breast nurse and a radiation oncologist) always evaluated skin toxicity. During follow-up visits, the following toxicities were also assessed: breast pain, as presence or absence, without differentiating for pain intensity; the presence of liponecrosis through ultrasound examination was also recorded; the lung toxicity was assessed with a thorax radiography requested as part of follow-up every 12 months and as presence of respiratory symptoms. Heart toxicity was evaluated only for symptomatic patients.

Dosimetric evaluation was based on DVH analysis of targets and OARs. Data were reported as mean doses, V_x _(volume receiving more than x dose) and D_y_ (dose received by at least y volume).

Statistical analysis and data correlation was conducted with the SPSS software (version 21.0). Standard descriptive statistics (mean standard deviation and cross tabulation analysis) was used to describe the data general behaviour. Univariate analysis was performed to investigate the prognostic role of individual variables, using ANOVA statistics for correlations with 0.05 as significance value.

## Results

**Fig. 1:**
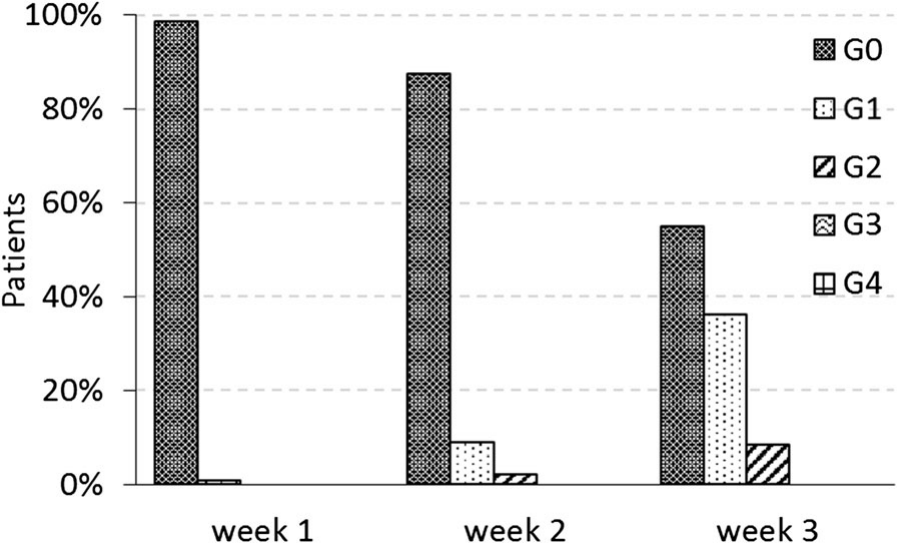
Acute skin toxicity during the radiotherapy treatment of three weeks

To date, among the 840 patients treated according to the phase II protocol, 144 had at least 2-year follow-up. The results presented in the current work refer only to this sub-group of patients. All patients have an invasive carcinoma (no patients with DCIS were present in this subset due to their late enrolment). The median follow-up was 37 months (range 24–55 months). Patient characteristics are summarized in Table 1. In the currently analysed group, four patients had synchronous bilateral breast treatment. Acute skin toxicities reported during the treatment course at one, two, and three weeks, are reported in Fig. 1, where a maximum of G2 acute toxicity was reported at the third week of treatment by 8 % of patients. No patient with G2 acute toxicity had moist desquamation. No grade higher than G2 was reported.

**Table 1:** Patient characteristics

Number of patients	144
Age [years old]	
Median [range]	62 [30, 88]
Mean ± SD	60 ± 11
Breast laterality	
Left	71 (49.3 %)
Right	69 (47.9 %)
Bilateral	4 (2.8 %)
Performance Status	
0	134 (93.1 %)
1	10 (6.9 %)
2	0
Unknown	0
Histology	
Ductal infiltrating ca.	123 (85.4 %)
Lobular infiltrating ca.	14 (9.7 %)
Other	5 (3.5 %)
DCIS	0
Unknown	2 (1.4 %)
Grading	
G1	16 (11.1 %)
G2	102 (70.8 %)
G3	22 (15.3 %)
Unknown	4 (2.8 %)
ER	
ER = 0	6 (4.2 %)
ER > 5	135 (93.8 %)
Unknown	3 (2.1 %)
PgR	
PgR = 0	13 (9.0 %)
PgR > 5	128 (88.9 %)
Unknown	3 (2.1 %)
KI67	
KI67 ≤ 20	103 (71.5 %)
KI67 > 20	36 (25.0 %)
Unknown	5 (3.5 %)
cerbB2	
Negative	114 (79.2 %)
Positive	19 (13.2 %)
Unknown	11 (7.6 %)
Surgical margins	
Negative	128 (88.9 %)
Close	4 (2.8 %)
Positive	1 (0.7 %)
Unknown	11 (7.6 %)
Lesion diameter	
≤ 1 cm	39 (27.1 %)
Between 1 and 2 cm	68 (47.2 %)
≥ 2 cm	25 (17.4 %)
Unknown	12 (8.3 %)
pT	
1	1 (0.7 %)
1a	4 (2.8 %)
1b	36 (25.0 %)
1c	77 (53.5 %)
1mic	2 (1.4 %)
2	23 (16.0 %)
X	0
is	0
Unknown	1 (0.7 %)
pN	
0	122 (84.7 %)
1a	12 (8.3 %)
1sn	5 (3.5 %)
N1(mi)	1 (0.7 %)
X	4 (2.8 %)
Unknown	0
Chemotherapy	
Yes	21 (14.6 %)
No	123 (85.4 %)
Hormonotherapy	
Yes	120 (83.3 %)
No	24 (16.7 %)

**Fig. 2:**
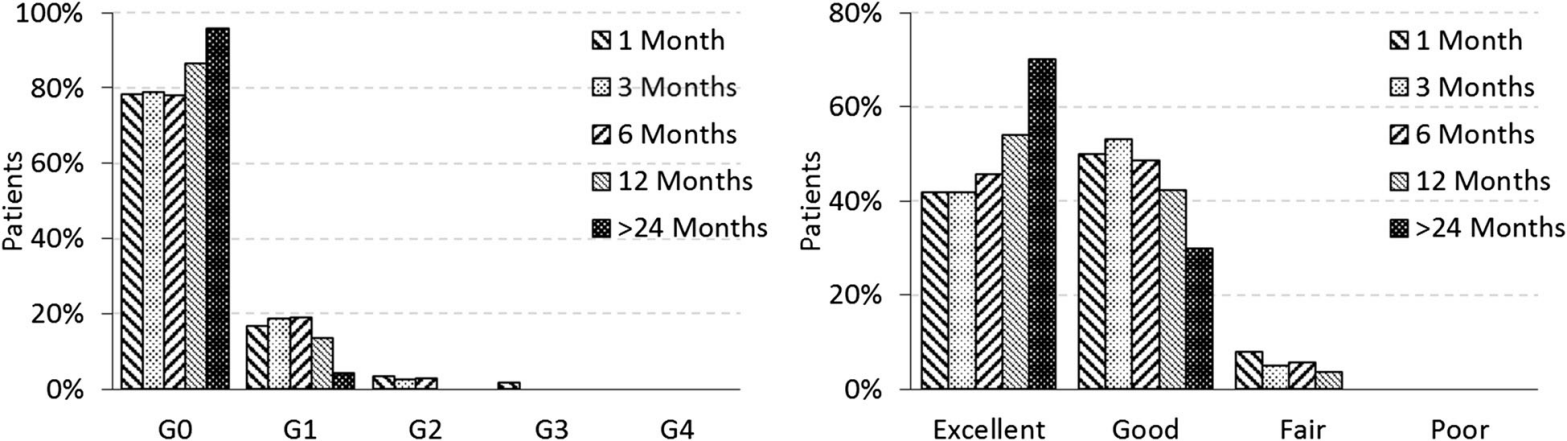
Skin toxicity and cosmetic outcome after the radiotherapy treatment

Skin toxicity and cosmetic results after the treatment are shown in Fig. 2 at 1, 3, 6, 12 months after radiotherapy and at the last follow-up (>24 months, median 37). One single case of G3 toxicity was recorded at 1-month follow-up visit. It related to a patient treated for bilateral breast cancer without chemotherapy, while hormonotherapy (tamoxifen and aromatase inhibitors) was administered. This high toxicity disappeared at the next follow-up at 3 months; the same patient developed no skin toxicity during the treatment. At one year, the highest reported skin toxicity was G1, as dermatitis, in 14 % of the patients, while in 4 % at the last follow-up, showing an almost complete recovery of the morbidity.

A correlation was found between late skin toxicity (recorded from 6 months after radiotherapy), as hyperpigmentation, and breast volume receiving 40.5Gy (*p <* 0.0001). At one-year follow-up, the group of patients presenting no skin toxicity had PTV_WB volume of 591 ± 39 cm^3^, while in patients presenting mild late toxicity (G1) this was 1105 ± 98 cm^3^, i.e. almost doubled. At the last follow-up, given the small number of G1 toxicity, the correlation decreased to *p =* 0.014, keeping however the same trend (PTV_WB volumes of 646 ± 36 and 1104 ± 198 cm^3^ for no and mild toxicity, respectively). As late skin toxicity, no patients presented fibrosis nor teleangiectasia.

Concerning pulmonary toxicity, diffuse reticular accentuation of the interstitium at chest radiograph was recorded at the last follow-up in 36 patients (25 % of the study population). We could consider this radiological finding as a G1 of pulmonary fibrosis according to CTCAE v. 4.0, but no further examination as chest CT was undertaken to confirm the data. No correlation was found between this feature and the dosimetric results, and no patients developed respiratory symptoms.

No cases of heart toxicity were recorded until the last follow-up.

Breast pain was present, at the last follow-up (median 37, range 24–55 months), in 3.5 % of the patients. This figure changed during follow-up: from 6 to 12, 12 to 24, 24 to 36, and more than 36 months after radiotherapy it was present in 21.6 %, 12.2 %, 6.2 % and 5.3 % of the analysed cases, showing an improvement of breast pain with time.

Liponecrosis, when present, was reported in the boost region (higher dose/fraction region). It developed in 23.4 % of the patients (as recorded at last follow-up). Patients presenting liponecrosis had PTV boost volume of 48 ± 8 cm^3^ (as mean ± standard error of the mean), while it was of 37 ± 4 cm^3^ for patients not presenting liponecrosis; correlation between liponecrosis and boost volume was however not statistically significant.

As clinical outcome, 143 patients (99.3 %) had no recurrence or metastatic disease. One patient (0.7 %) developed liver metastases at 39 months from radiotherapy.

**Fig. 3:**
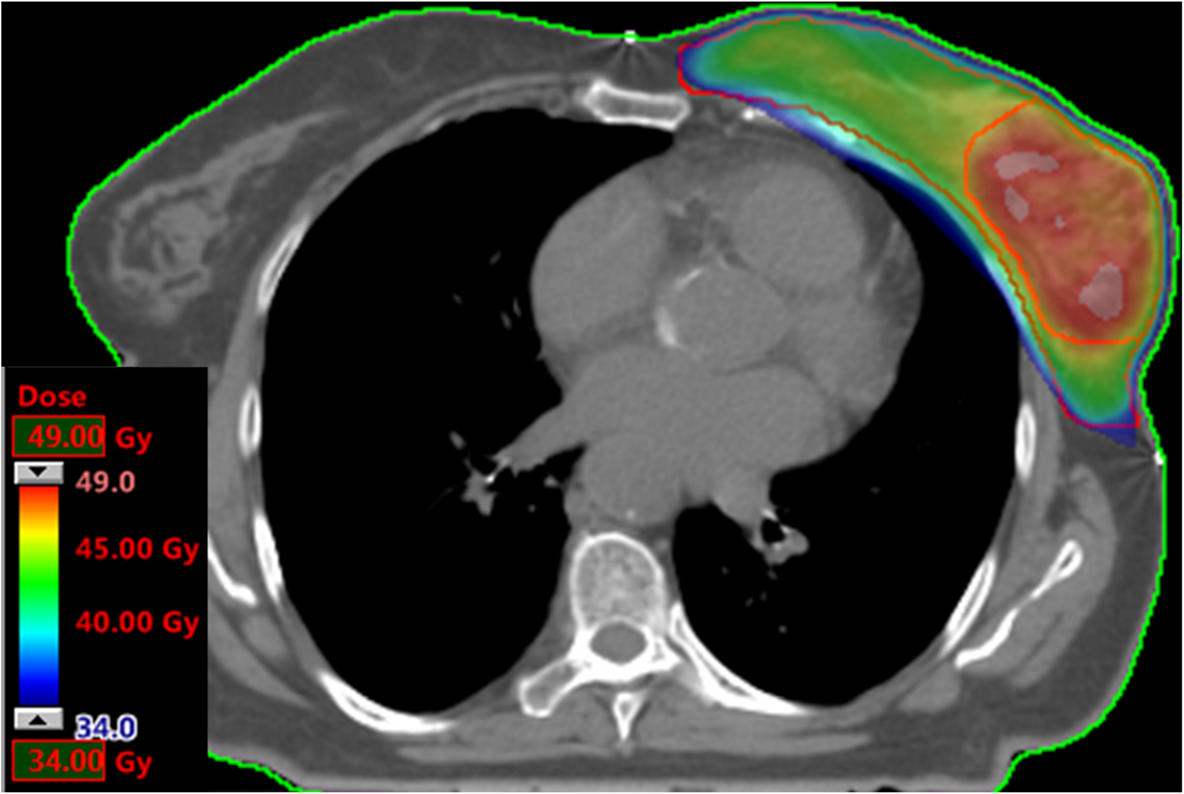
Dose distribution of a typical breast treatment with VMAT-SIB

Concerning dosimetric results, in Table 2 a summary of some parameters related to the main OARs is reported. All the dosimetric objectives were fulfilled. In the same Table 2 also the dose homogeneity inside the two targets is presented as standard deviation parameter. In Fig. 3 a typical dose distribution is shown.

**Table 2:** Dosimetric results, as average ± SD over all patients

Structure	Parameter	All patients	Left side breast patients	Right side breast patients
Lung, ipsilateral	Mean [Gy]	7.6 ± 1.5	7.5 ± 1.2	7.7 ± 1.7
	V_20Gy_ [%]	7.6 ± 2.7	7.4 ± 2.0	7.7 ± 3.2
	V_5Gy_ [%]	50.9 ± 14.7	50.2 ± 13.3	51.7 ± 15.9
Lung, contralateral	Mean [Gy]	2.5 ± 1.3	2.4 ± 0.9	2.6 ± 1.6
Heart	Mean [Gy]	5.1 ± 2.1	6.0 ± 1.6	4.0 ± 2.1
	V_18Gy_ [%]	1.3 ± 1.7	2.3 ± 1.8	0.2 ± 0.4
	V_40Gy_ [%]	0.0 ± 0.0	0.0 ± 0.0	0.0 ± 0.0
Breast, contralateral	Mean [Gy]	2.3 ± 0.6		
Skin	Mean [Gy]	22.7 ± 4.3		
PTV_boost	St.Dev. [Gy]	0.9 ± 1.0		
PTV_WB	St.Dev. [Gy]	1.5 ± 0.4

## Discussion

The key point of the present study is the clinical application of the hypofractionated-SIB scheduling using VMAT technology in breast treatments, firstly on the toxicity evaluation.

The large randomized trials on hypofractionation did not explore the integration of tumour bed boost [7, 9–11]. The Canadian trial [7] had no boost and the UK trials [9–11] delivered a sequential boost dose increasing in overall treatment time. Thus, no definitive conclusions on this issue came from these studies.

Various single-institutional experiences are published concerning boost association. Two analyses have studied the association of concomitant boost and the 3-week course of radiation. Chadha et al. [23] analyzed data from 160 early breast cancer patients and reported minimal acute toxicity (G1 skin effects in 70 % and G2 skin effects in 5 % of the patients), no late toxicity higher than G2 (among patients with > 2 years follow-up) and excellent clinical outcomes at a median follow-up time of 3.5 years (local relapse-free survival rate was 99 %). They used field-in-field technique to optimize a total dose of 40.5 Gy in 2.7 Gy fractions on WB with a concomitant boost of 4.5 Gy in 0.3-Gy fractions. Similar results using a similar treatment scheme were reported by Formenti et al. [24], analyzing data from 91 patients treated with IMRT in prone position.

In our experience, the use of SIB revealed optimal patient compliance, without a significant increase of skin toxicity or breast pain, as other studies previously described have confirmed [24–26]. One single case of G3 toxicity was recorded at 1 month by the end of treatment and disappeared at 3 months of follow-up. This good toxicity profile is expected, as with the SIB technique, differently from sequential boost schemes, there is no excess dose outside the tumour bed PTV, as the two dose levels are planned together to be homogeneous during the plan optimization. This fact has been analysed by Franco et al. [27] in their phase II trial with tomotherapy treatments.

We also evaluated the presence of liponecrosis as recorded from ultrasound exam. When present, liponecrosis was reported in the boost region (higher dose/fraction region). To our knowledge, few published papers report this data, mainly related to the use of Intraoperative Radiotherapy (IORT) combined to oncoplastic surgery [28, 29]. In our group of patients, we find no real correlation between breast pain and liponecrosis, while an interesting feature was the progressive improvement of breast pain with time during the follow-up.

To date, there are two large phase III prospective trials to compare sequential boost vs. concomitant boost. The RTOG 1005 trial is a phase III prospective trial comparing conventional radiotherapy (50 Gy in 25 fractions or with hypfractionation option of 42.7 Gy in 16 fractions) followed by a sequential boost of 12–14 Gy in 6–7 fractions vs. a hypofractionated accelerated WBRT schedule of 40 Gy in 15 fraction with a concomitant boost to the tumor bed up to 48 Gy in the same 15 fractions. The IMPORT High trial tests dose-escalated RT delivered with IMRT in early breast cancer patients with higher than average risk of local recurrence. The standard arm comprises 40.5 Gy in 15 fractions and a sequential tumor bed boost of 16 Gy in 8 fractions; as regards experimental arms, for 15 fractions treatments, the first arm received fractions of 2.4 Gy, 2.67 Gy and 3.2 Gy to the whole breast, the index quadrant and the tumour bed, respectively, while the second arm receives fractions of 3.53 Gy to the tumour bed [30]. Both these trials have been closed to accrual and results will provide evidence on this debated issue.

Recently, Bartelink et al. [31] published mature data on the eff ect of a radiation boost of 16 Gy on overall survival, local control, and fibrosis for patients with stage I and II breast cancer compared with patients who received no boost. The results support the use of boost for younger patients confirming a significant improvement in local control (not in overall survival). The relative benefit of the extra radiation dose for local control was independent of age, but with increasing age the absolute gain in local control decreased. Therefore, the authors concluded suggesting the possibility to avoid boost in most patients older than age 60 years. Notwithstanding, in the present study we also included patients over 60 years. Currently, the ideal radiotherapy approach for ederly patients with low risk early stage breast cancer is under investigation. Different personalized treatments could be proposed for these patients, such as Accelerated Partial Breast Irradiation (APBI) [32], highly hypofractionated schedules (FAST Trial or FAST-Forward Trial) [33], hormonal therapy only [34], or surgery without other adjuvant treatments. Surely, we need a study to compare these different approaches identifying the best therapeutic strategy for this subgroup of patients.

Over the past decade, literature data concerning the use of hypofractionated WBRT in terms of efficacy and safety have been steadily increasing. In particular, the analysis of 10-year outcome of START-B trials [11] reported the rate of local tumour relapse of 3.8 % in the hypofractionated arm compared with 5.2 % in conventional fractionation group. Bane et al. [35] analysed tumour factors predictive of response to hypofractionated radiotherapy. They concluded that patients with node-negative breast tumours of all grades and molecular subtypes (luminal A, luminal B, HER2 enriched and basal-like) might be safely treated with accelerated regimens because these features and hypoxia did not predict response to hypofractionation.

In our study, despite the short follow-up time, we showed no cases (on 144 patients) of local tumour recurrence, as the only evidence of disease was a metastatic expression after more than two years.

Two of the most debated topics about the use of hypofractionation schemes are the association with systemic therapy (neoadjuvant or adjuvant) and the consequences of this schedule in young patients. There are some experiences with encouraging but inconclusive results, and we probably need longer follow-up data to confirm the role of these schedules especially in young women. In our group of patients only 11 were less than 45 years old and 21 underwent chemotherapy, for whose toxicities have been scored similarly to the other analysed patients.

As regards hypofractionation and large breasts we have more consistent data [36]. Hannan et al. [37] evaluated the effects of breast size on clinical toxicity in hypofractionated WBRT using intensity modulation. Their schedule had an acceptable toxicity profiles irrespective of breast size. In our group of patients, we found a correlation between late skin toxicity and the breast volume receiving 40.5Gy. The difference was evaluated in terms of G0/G1 skin toxicity, while no grade 2 of skin toxicity was recorded. One of the main reasons of the radiation oncologists‘reluctance towards the use of accelerated schedules is the unknown impact of a higher daily dose/fraction on late toxicity. Evidence from laboratory and clinical studies suggests that fraction size has a larger impact on late effects than acute effects of radiotherapy, mainly for a potentially increased risk of cardiac or lung toxicity.

Darby et al. [38], for conventional fractionation schemes, correlated the risk of ischemic heart disease with dose, estimating an increased risk of 7.4 % per Gy of mean heart dose, knowing that it is related to larger heart volume included in the two tangential fields. In our study, by using VMAT technology, no cardiological events were recorded, but we did not request particular diagnostic exam to evaluate cardiological function without clinical manifestation. Although at such short follow-up, the result of no cardiac events is reassuring. However, it does not guarantee that these patients are not at elevated risk of cardiac events over a longer follow-up period. A longer follow-up is needed in this respect.

Few studies investigated pulmonary toxicity in the hypofractionation group. Van Parijs et al. [39] studied the feasibility of a 3-week accelerated schedule for 70 stage I–II breast cancer patients. Pulmonary function was evaluated by FEV1 change or DLco; at 2 years, the reduction was seen greater in patients who had undergone conventional fractionation compared with hypofractionation arm. Patients in that study received tomotherapy treatment, i.e. a dose distribution inside the lung similar to what is shown in our present study. Our patients had thorax X-ray examination every 12 months as part of their follow up programme. In 25 % of the patients we recorded a diffuse reticular accentuation of the interstitium. However, no patients developed pneumonitis nor respiratory symptoms until the last follow up.

Our study present however two limitations: the rather short follow-up (at least compared to the time needed to express very late toxicities), and the quite limited number or patients. However the results presented here are encouraging enough to continue in the exploration of this field. For that reason, it is our goal to continue to follow this same cohort of patients; they will be re-evaluated after a longer follow-up, especially focusing on the heart and lung toxicity, as well as local control.

## Conclusion

Hypofractionated VMAT-SIB radiotherapy delivered in 3 weeks showed encouraging 2-years toxicity and clinical results for early stage breast cancer treatment. A longer follow-up is needed to confirm these data.

## Abbreviations

AJCC: American joint committee on cancer
APBI: Accelerated partial breast irradiation
ASTRO: American societry for radiation oncology
BCS: Breast conserving surgery
CBCT: Cone beam CT
CT: Computed tomography
CTCAE: Common terminology criteria for adverse events
CTV: Clinical target volume
DCIS: Ductal carcinoma in situ
DVH: Dose volume histogram
IMRT: Intensity modulated radiotherapy
MLC: Multileaf collimator
OAR: Organ at risk
OTT: Overall treatment time
PTV: Planning target volume
RTOG: Radiation therapy oncology group
SIB: Simultaneous integrated boost
VMAT: Volumetric modulated arc therapy
WB: Whole breast
WBRT: Whole breast radiotherapy
